
JOURNAL INFORMATION
==============================
NLM Title Abbreviation: Open Forum Infect Dis
Journal ID: Open Forum Infect Dis
NLM Journal ID: 101637045
Journal ID: ofid

Open Forum Infectious Diseases

EISSN: 2328-8957
Publisher: Oxford University Press

ARTICLE INFORMATION
==============================
PMCID: PMC10632715
PMID: 37953816
DOI: 10.1093/ofid/ofad529
Article ID: ofad529
Article version: 1
Subjects: Insight Article, AcademicSubjects/MED00290

The Importance of a Broad Consortium of Bacteria for a Healthy Gastrointestinal tract: A Narrative Review of the Live Biotherapeutic Product REBYOTA

https://orcid.org/0000-0002-2221-008X Tillotson Glenn GST Micro LLC, North, VA, USA

Vedantam Gayatri School of Animal & Comparative Biomedical Sciences, University of Arizona, Tucson, AZ, USA

Shin Jae Hyun Division of Infectious Diseases & International Health, University of Virginia, Charlottesville, VA, USA

Warren Cirle A Division of Infectious Diseases & International Health, University of Virginia, Charlottesville, VA, USA

corresponding author - gtillotson@gstmicro.com
Publication date: 2023 Nov
Electronic publication date: 2023 Nov 6
Volume: 10
Issue: 11
Electronic Location ID: ofad529
2023 Nov 6
Copyright: © The Author(s) 2023. Published by Oxford University Press for the Infectious Diseases Society of America.
Copyright year: 2023
License: This is an Open Access article distributed under the terms of the Creative Commons Attribution License (https://creativecommons.org/licenses/by/4.0/), which permits unrestricted reuse, distribution, and reproduction in any medium, provided the original work is properly cited.
License URL: https://creativecommons.org/licenses/by/4.0/

Keywords: Clostridioides difficile, microbiome, broad consortium, foundational species, keystone species
Page count: 8

==============================

Open Forum Infectious Diseases

Open Forum Infectious Diseases 2023; 10: 11
doi: 10.1093/ofid/ofad529

InSight

### The Importance of a Broad Consortium of Bacteria for a Healthy Gastrointestinal tract: A Narrative Review of the Live Biotherapeutic Product REBYOTA

Glenn Tillotson* [1], Gayatri Vedantam [2], Jae Hyun Shin [3], Cirle A. Warren [3]

[1] GST Micro LLC, North, VA, USA (*corresponding author – gtillotson@gstmicro.com)
[2] School of Animal & Comparative Biomedical Sciences, University of Arizona, Tucson, AZ, USA
[3] Division of Infectious Diseases & International Health, University of Virginia, Charlottesville, VA, USA

**Keywords:** *Clostridioides difficile*, microbiome, broad consortium, foundational species, keystone species.

### Abstract

The human microbiome comprises an estimated 100 trillion organisms and their genetic content which consists of bacteria, viruses, fungi, and parasites. There are more than 1,000 different species of bacteria in a healthy gut with the majority residing in the lumen of the large intestine. The balance of these organisms is vital for the balance of health and the relative abundance is essential to provide the various metabolites and other chemicals necessary for normal microbial environment which is known as eubiosis. When the balance or relative abundance is shifted, a disturbance of metabolites can lead to a condition, dysbiosis. Certain organisms are responsible for essential metabolites such as short chain fatty acids or bile acids. These molecules have various consequences including disruption of colonization resistance or lack of GABA production which has been shown to affect the gut-brain axis. It is the relative amounts of certain taxa or species that ensure this balance among these various organisms constitute a broad consortium. This consortium comprises Bacteroidia and Firmicutes which includes Clostridium species. Herein we describe the important features of various organisms and their metabolites.

### Introduction

Darwin summarized the complexity of the living world in "On the Origin of Species". He hypothesized about a "tangled bank", inhabited by a multitude of species of plants, birds, insects, and worms, all "dependent upon each other in so complex a manner" [1]. Only now are we beginning to explore these same complexities in the microbial world. Like Darwin's tangled bank, microbial ecosystems include not only the remarkably high number of species inhabiting diverse niches, but also from the many ways in which these species interact. The array of taxa identified in various niches is considerable with the most species identified in the gut while microbiomes have been identified in every other niche from the placenta, skin, lung, mouth and even the brain. Often these microbiomes evolve over time. As new 'omic' methods are developed, our understanding of the changes in these niches will improve our ability to identify key organisms in the healthy environment [2].

The term 'microbiota' refers to the roughly 1100 species of bacteria, archaea, microeukaryotes, and viruses that share the human body space, and function in commensal, symbiotic, or pathogenic relationships [3, 4]. The dominant bacterial phyla in the gastrointestinal (GI) tract include Bacteroidetes (or Bacteroidota) and Firmicutes, followed by Actinobacteria and Proteobacteria [5-7].

It is important to note that the various microbial taxa do not exert equivalent impacts on the GI ecosystem. The GI tract can be considered a functionally redundant space whereby the functioning of the system may remain unaffected by the loss of some species due to compensation by other similarly functioning organisms. This resilience and stability of the gut microbiome is essential to human health and development.

In contrast, foundational and keystone species in the GI tract have unique and influential roles [8]:

- A foundational species is one that dictates the structure of the ecosystem by creating stable conditions for other species, and by modulating and stabilizing fundamental processes.
- A keystone species is vital for maintaining the organization and diversity of microbial consortium via biotic and abiotic interactions with community members. These often modestly

© The Author(s) 2023. Published by Oxford University Press for the Infectious Diseases Society of America. This is an Open Access article distributed under the terms of the Creative Commons Attribution License (https://creativecommons.org/licenses/by/4.0/), which permits unrestricted reuse, distribution, and reproduction in any medium, provided the original work is properly cited.

**Figure 1** Specific functions of Bacteroidetes and Firmicutes

### Bacteroidetes

1 **Sphingolipids** are membrane components and important structural as well as signaling molecules that regulate inflammation and immunity. A variety of Bacteroidetes-derived sphingolipids including ceramide phosphoinositol and deoxy-sphingolipids have been identified. These sphingolipids are critical for maintaining homeostasis and symbiosis in the gut [18].

2 Gut microbes including Bacteroidia can produce metabolites with high neuroactive potential (neurotransmitters), including **γ-aminobutyric acid (GABA)**, the major inhibitory neurotransmitter in the brain. Multiple *Bacteroides sp* strains produce GABA through a highly conserved glutamate decarboxylase (GAD)-system composed of a GAD, a glutaminase, a glutamate/GABA antiporter, and a potassium channel. All four genes of the GAD-system are present in 90% of *Bacteroides* genomes. *B. ovatus* synthesizes GABA and modulates the levels of tryptophan and glutamine [19, 20].

3 The **type VI secretion system (T6SS)** The type VI secretion system (T6SS) is a molecular conduit that enables Gram-negative bacteria the ability to translocate effector proteins directly into neighboring cells. The T6SS in Bacteroidetes provides an important contribution to colonization resistance and commensal stability [21].

4 **Bacteroidetocins** are a family of broad-spectrum peptide toxins produced by Bacteroidetes species that have inhibitory activity against diverse *Bacteroides* and *Parabacteroides* species, including environmental, symbiotic, and pathogenic members [22].

5 ***Bacteroides uniformis*** encodes structurally and functionally distinct Β-glucuronidases with specific roles in processing drug-glucuronide and glycan substrates, highlighting the broad structural and functional diversity among these enzymes within a single human gut microbe [23].

6 ***Bacteroides vulgatus FTJS7K1*** was found to protect against lipopolysaccharide-induced acute intestinal injury via modulation of cytokine production in the colon tissue and regulation of the structure of the gut microbiota [24].

7 ***Bacteroides thetaiotaomicron*** administration has been found to inhibit the colonization of *Clostridioides difficile* and inhibit inflammation in the colon by attenuating lymphocyte infiltration in the mucous layer and decreasing edema in the submucosa [25].

8 The polysaccharide capsule of *B. thetaiotaomicron* plays an important role in **colonization resistance**, suppression of toxin release and production of Β-lactamases which can maintain the balanced microbiota [26-28].

9 ***Parabacteroides distasonis*** has been found to alleviate obesity and metabolic dysfunctions via production of succinate and secondary bile acids (lithocholic acid and ursodeoxycholic acid) in the gut [29].

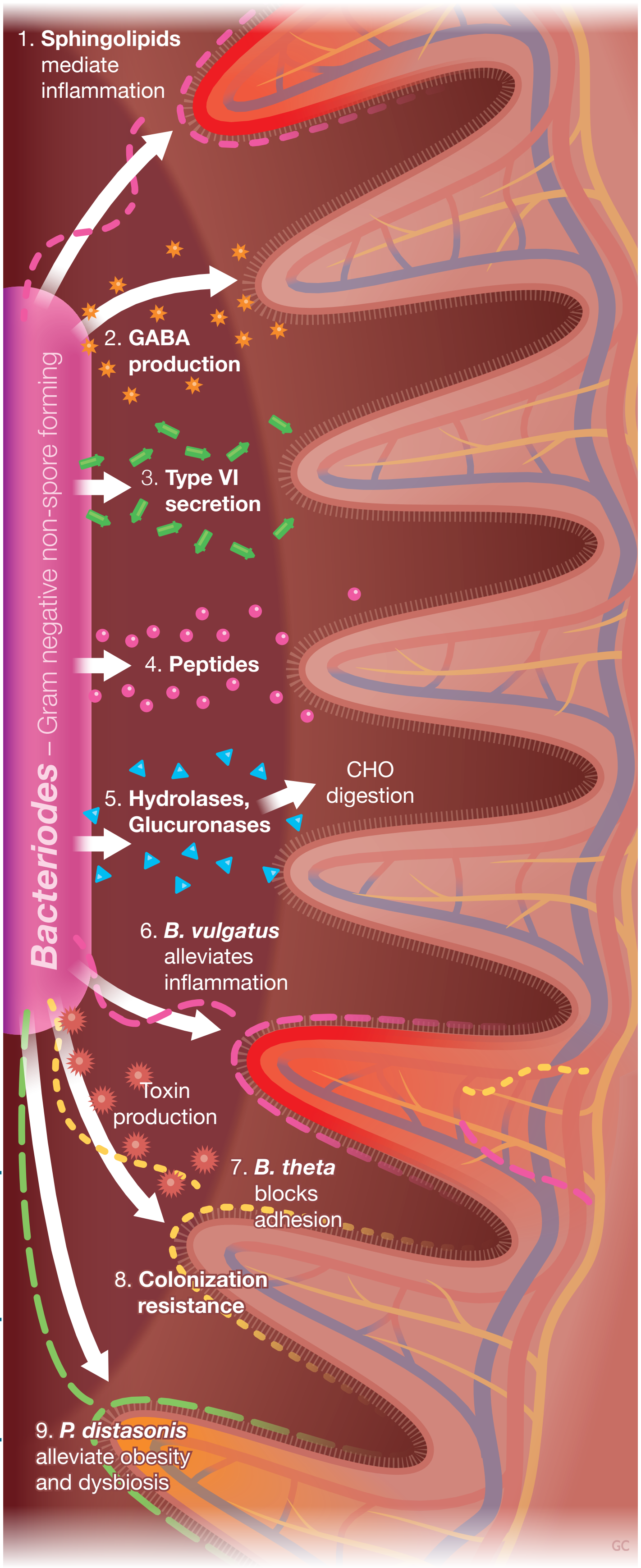

### Firmicutes

1 The spore-forming clusters IV and XIVa (also known as *Clostridium leptum* and *coccoides* groups, respectively) control **CD4$^+$ T regulatory cells** ($T_{regs}$), which play a critical role in the maintenance of immune homeostasis [30].

2 ***C. scindens***, C. scindens, a member of the *Clostridium* XIVa clade, has also been shown to produce butyrate, which plays a role in colitis resolution, and secretes the tryptophan-derived antibiotic, 1-acetyl-β-carboline that inhibits multiple gram-positive pathogens found in the gut, including *Clostridioides difficile* [31, 32].

Firmicutes exhibited a positive correlation with serum total testosterone levels independent of host factors (e.g., age, body mass index, triglyceride, and total cholesterol). Therefore, Firmicutes may play a role in testosterone metabolism [33].

### Other

*Akkermansia muciniphila* is a gram-negative anaerobe that decreased serum aspartate transaminase and alanine transaminase, alleviated liver histopathological damage, substantially attenuated serum levels of pro-inflammatory cytokines and chemokines, significantly decreased hepatocellular apoptosis, and strengthened intestinal barriers and reduced systemic LPS level. *A. muciniphila* also increased the diversity and richness of the microbial community and simultaneously modulated the varieties of gut microbes, such Lachnospiraceae and Ruminococcaceae [34].

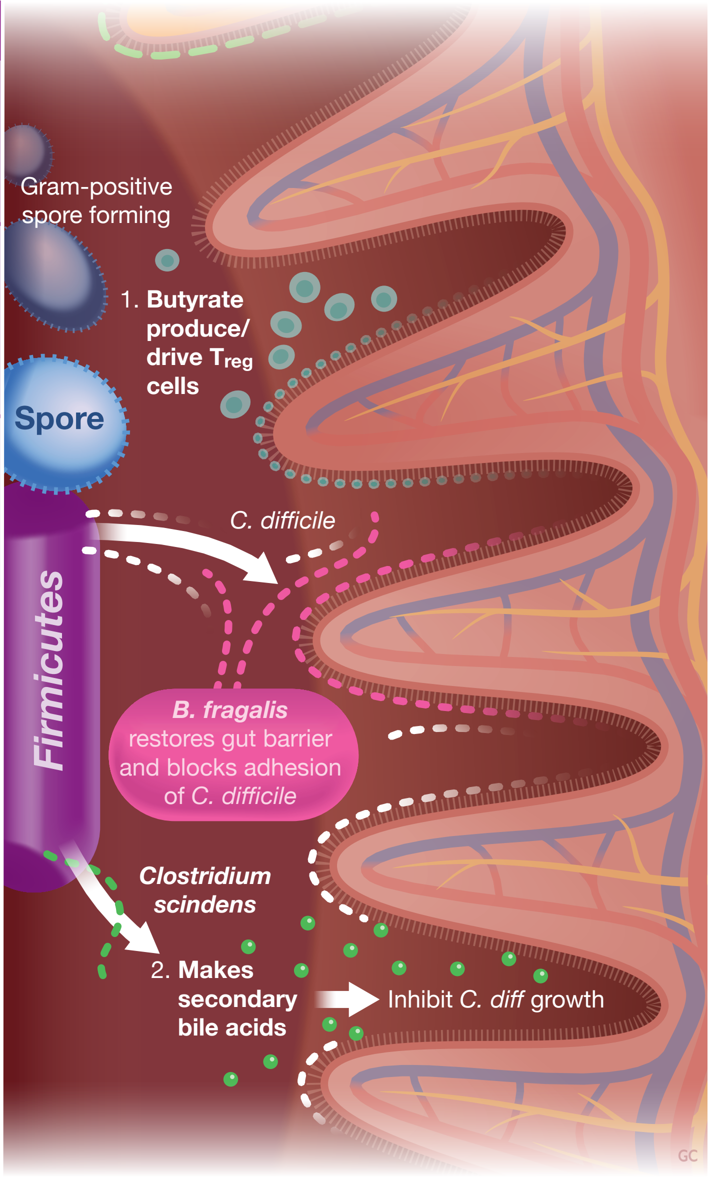

abundant species underscore vulnerabilities in the homeostasis of the ecosystem since their abrogation can result in a loss of biodiversity as well as key functions

Trosvik and Mulnick (2015) used multiple genomic approaches to demonstrate connectedness in the ecological network as a proxy for a taxon's importance in ecosystem dynamics. Actinobacteria fit the description of a "keystone taxon" due to its high degree of ecological connectedness and positive influence within the community despite its low relative abundance [8].

While the above organisms are involved in interactions that stabilize the ecosystem via metabolic, immunologic, and physiologic impacts, they are not the only beneficial species. A complex and diverse microbiota also includes Bifidobacteria, Prevotella, Akkermansia, and members of Firmicutes such as the Ruminococcaceae, Lachnospiracaea, and Clostridiales [9, 10]. Therefore, a broad consortium of bacterial phyla is reflective of a healthy, complex microbiome. Indeed, these communities in aggregate may be referred to as a metabolic organ with crucial functions that exert wide-ranging impacts on human health.

The foundation taxon, Bacteroidetes, has a very broad metabolic and physiologic potential that includes immunomodulatory effectsas well as the ability to adapt and persist in changing gut environments, thus contributing to the stability of the microbiome [8, 11, 12]. Firmicutes, the most abundant and diverse gut species [13] has functions that include anti-inflammatory effects [14, 15] and fortification of the gut barrier [16]. Both of these phyla are key producers of short chain fatty acids (SCFAs), an important energy source for host intestinal cells that constitute the gut barrier and secrete vital immunomodulatory factors. Multiple species in the Bacteroidetes phylum produce propionate and acetate, while Firmicutes produce propionate and butyrate [17]. **Figure 1** illustrates examples of relevant functions of these keystone species in maintaining intestinal homeostasis.

In essence, a "healthy" microbiota requires a diverse consortium of microbial organisms, including both Firmicutes and Bacteroidetes and other key organisms that in concert support vital functions of the intestinal tract. The specific microbial composition likely differs from individual to individual as this is influenced by various host factors such as diet, lifestyle, comorbidities, medications, and others. Systemic or local conditions that skew the distribution or functioning of the microbial community (or a state of dysbiosis) may increase susceptibility to certain diseases.

### *Clostridioides difficile* infection (CDI)

*Clostridioides difficile* infection (CDI) is the most common cause of antibiotic- and healthcare-associated infectious diarrhea in the United States [35, 36], associated with almost half a million infections and 29,300 deaths annually [35, 37]. CDI imposes substantial clinical, psychological, social, and professional burdens [38], with some studies estimating its attributing inpatient treatment and mitigation costs to be as high as $5 billion annually [39]. The causative agent, *C. difficile*, is a fastidiously anaerobic, Gram-positive, spore-forming bacterium; toxin-producing (toxigenic) strains can produce up to three exotoxins, Toxin A, Toxin B, and binary toxin [40].

The incidence of recurrent CDI (rCDI) has also increased significantly in recent years, and this has been identified as a major public health challenge [35, 41-44]. Especially in the elderly, up to 35% of patients who initially respond to antimicrobial therapy experience disease recurrence, of which 60% experience subsequent recurrences [45].

CDI is characterized by a disrupted microbiome [46-48] that includes decreased Bacteroidetes and Firmicutes phyla [13, 49-52], which play a significant role in protecting against the disease. Disruption of the gut microbiome, especially common following antibiotic treatment, and consequent disruption of bile acid homeostasis, creates an ideal environment for *C. difficile* colonization [53-55]. Specifically, *B. thetaiotaomicron* positively impacts bile acids that inhibit *C. difficile* growth and reduce spore germination [25]. *Bacteroides uniformis* and members of the *Lachnospiraceae* and *Ruminococcaceae* families (i.e., *Clostridium* clusters XIVa and IV), including *Clostridium scindens*, are known/predicted to have 7α-dehydroxylation activity, which transforms primary bile acids to secondary bile acids [56, 57]. These secondary bile acids inhibit spore germination, growth, and toxin activity of *C. difficile*. Additionally, conversion to secondary bile acids depletes the pool of primary bile acids, some of which induce spore germination [58-64].

*Bacteroides thetaiotaomicron* attenuates colonization of *C. difficile* and inflammation in the colon. It suppresses toxin release from *C. difficile* by inhibiting cell autolysis [27] and helps restore healthy microbial balance by increasing Bacteroidetes and lowering Proteobacteria levels [25].

*Bacteroides fragilis* prevents CDI by restoring the gut barrier (by eliciting robust ZO-1 and Muc-2 expression), inhibiting *C. difficile* adherence, attenuating the decrease in CDI-induced transepithelial electrical resistance, and improving commensal bacterial diversity and relative abundance [65]. Finally, members of the *Lachnospiraceae* and *Ruminococcaceae* families of *Clostridiales* order may harbor coprostanoligenic activity; this cholesterol-reducing effect suggests that these particular bacteria may play an important role not only in host lipid metabolism, but also susceptibility to CDI [66].

### Live biotherapeutic products

Identification of the GI tract organisms that potentiate gut health and provide colonization resistance against pathogens underpins emerging and novel therapies such as precision fecal microbiome transplantation (FMT). Currently, the consortium containing only spore-forming bacteria, VOWST (formerly SER-109), Seres Therapeutics) was FDA approved in April 2023 and the other select consortium, Vedanta candidate VE303 is in Phase III clinical trials.

The broad consortium human derived product, REBYOTA (RBL; Ferring Pharmaceuticals), was recently approved by the FDA. Uniquely RBL contains a wide range of bacterial species ($10^8$-$10^{10}$ organisms per dose) including >1x$10^5$ colony forming units (CFU) per mL of *Bacteroides* spp. Other organisms include members of the Firmicutes, *Faecalibacteria*, *Roseburia*, *Blautia*, and other non-spore-forming bacteria [67]. Recent data published from the large clinical program on RBL demonstrated good safety and efficacy along with replenishment of a healthy state of the diseased dysbiotic bowel associated with rCDI [68]. About 30% of patients had a first recurrence post index infection [69] and importantly, two studies followed patients for two years and no cases of bacteremia or fungemia were reported [70, 71]. Additionally the single dose group achieved a sustained clinical response of 95.7% over two years [72].

There is a totality of evidence from RBL- or placebo-treated participants in the randomized, double-blind, placebo-controlled trials PUNCH CD2 (NCT02589847; n = 153) and PUNCH CD3 (NCT03244644; n = 289, and from RBL-treated participants in the PUNCH OLS (NCT03931941; n = 653) that clinical response to RBL is associated with restoration of microbiome (**Figure 2**). Furthermore, clinical response to RBL is significantly associated with engraftment (**Figure 3**); the phylum *Bacteroidia* and the Firmicute phylum including the genus *Clostridia* showed the highest engraftment percentages in RBL-treated participants wherein 16/30 effective engrafters were *Bacteroidia* and nine were *Clostridia*. Specifically, 58% of *B. uniformis* species were characterized as effective engrafters [73].

### Summary key points

- The ecology of the healthy human Gl tract is dictated by a broad consortium of microorganisms including *Bacteroidia* as well as Firmicutes such as *Clostridium* species.
- Bacteroidetes modulate bile acid and SCFA metabolism to control *C. difficile* colonization, spore germination, and persistence.
- Bacteroidetes also exert other important functions including host immunity modulation, gut wall integrity, and general colonization-resistance.
- Firmicutes provide key metabolic pathways, such as SCFA metabolism, that augment the activities of Bacteroidetes.
- The broad consortium of microbiota is therefore a "metabolic organ" underpinning diverse aspects of human GI tract health.
- RBL is a broad consortium human derived product that demonstrates safety and efficacy up to two years with positive shifts in microbiota.

**Figure 2** Clinical response to RBL is associated with a microbiota shift reminiscent of a healthy gut consortium [73]

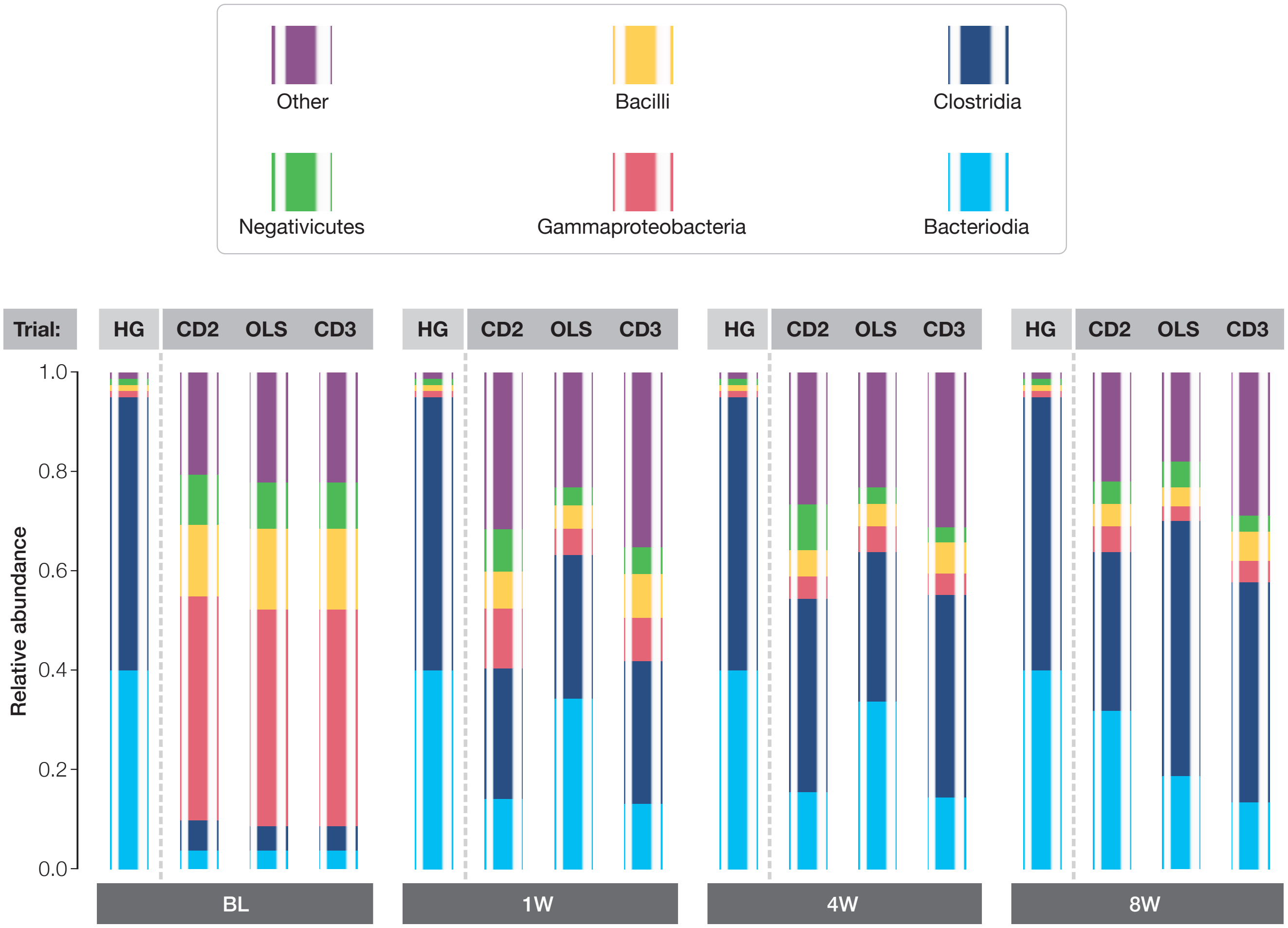

In an analysis of three trials (PUNCH CD2, PUNCH CD3, PUNCH OLS), baseline was characterized by predominance of *Gammaproteobacteria* and *Bacilli* whereas after treatment, microbiome composition shifted to predominance of *Clostridia* and *Bacteroidia* (healthy commensals), which occurred as early as week one. *Clostridium* species are Firmicutes.

Abbreviations: **HG**, Healthy gut; **CD2**, PUNCH CD2; **CD3**, PUNCH CD3; **OLS**, PUNCH OLS

**Figure 3** Bacteroidia and Clostridia have the highest engraftment percentages in RBL-treated participants [73]

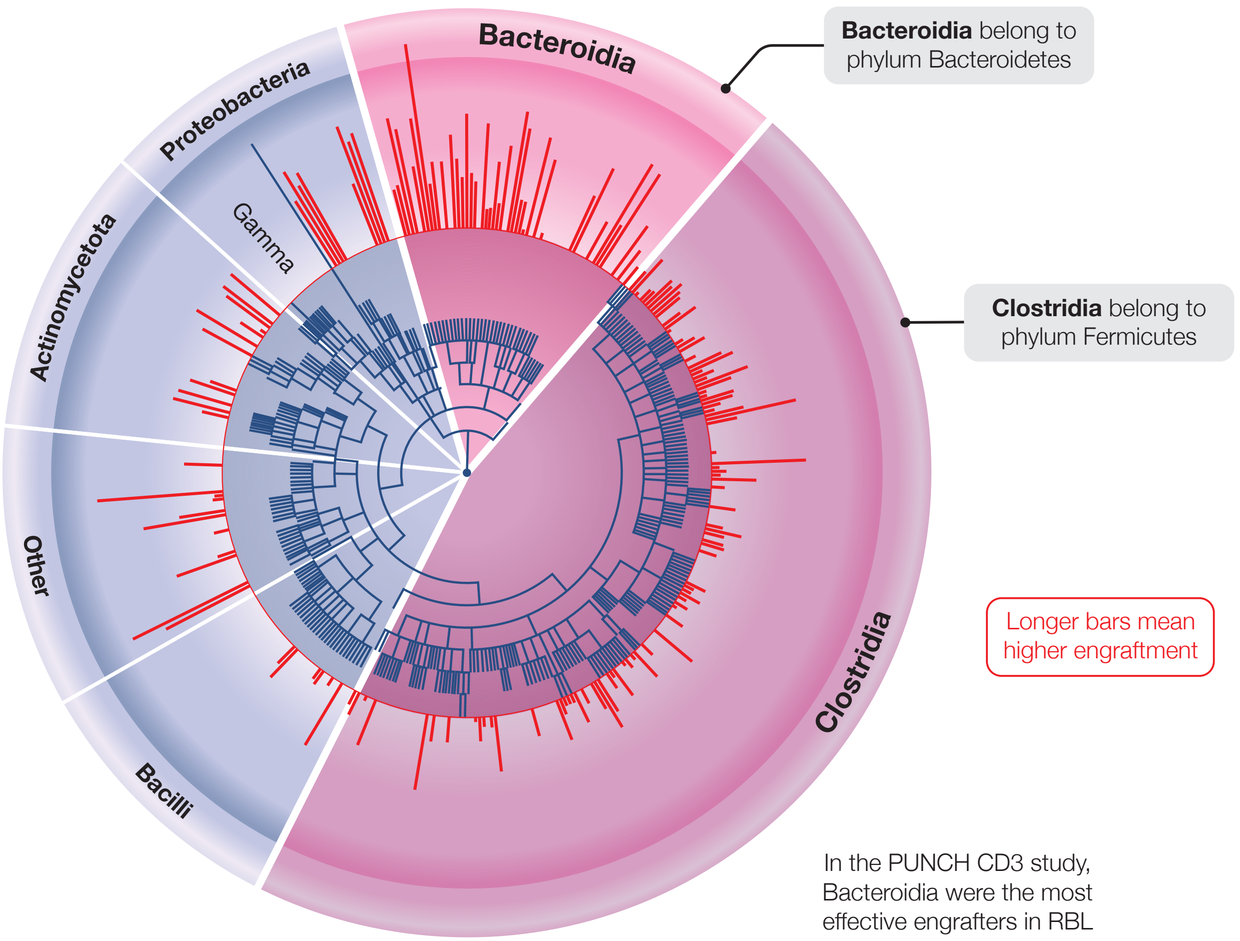

| | |
|---|---|
| Corresponding author name: | **Dr. Glenn Tillotson** GST Micro LLC, 327 Plantation Road, North, VA 23128, USA – email: gtillotson@gstmicro.com |

| | |
|---|---|
| InSight title: | **The importance of a Broad Consortium of Bacteria for a Healthy Gastrointestinal tract:** A Narrative Review of the Live Biotherapeutic Product REBYOTA |
| Sponsor: | This InSight was sponsored by **Ferring Pharmaceuticals.** |
| DOI: | https://10.1093/ofid/ofad529 |

OXFORD UNIVERSITY PRESS
Open Forum Infectious Diseases

© The Author(s) 2023. Published by Oxford University Press for the Infectious Diseases Society of America. This is an Open Access article distributed under the terms of the Creative Commons Attribution License (https://creativecommons.org/licenses/by/4.0/), which permits unrestricted reuse, distribution, and reproduction in any medium, provided the original work is properly cited.

### References

1. Darwin, C., On the origin of species by means of natural selection, or, The preservation of favoured races in the struggle for life. 1859, London: John Murray.
2. Hollister, E.B., C. Gao, and J. Versalovic, *Compositional and functional features of the gastrointestinal microbiome and their effects on human health.* Gastroenterology, 2014. **146**(6): p. 1449-58.
3. Qin, J., et al., *A human gut microbial gene catalogue established by metagenomic sequencing.* Nature, 2010. **464**(7285): p. 59-65.
4. Jimenez-Avalos, J.A., et al., *Classical methods and perspectives for manipulating the human gut microbial ecosystem.* Crit Rev Food Sci Nutr, 2021. **61**(2): p. 234-258.
5. Hillman, E.T., et al., *Microbial Ecology along the Gastrointestinal Tract.* Microbes Environ, 2017. **32**(4): p. 300-313.
6. Arumugam, M., et al., *Enterotypes of the human gut microbiome.* Nature, 2011. **473**(7346): p. 174-80.

7. Trosvik, P. and E.J. de Muinck, *Ecology of bacteria in the human gastrointestinal tract--identification of keystone and foundation taxa.* Microbiome, 2015. **3**: p. 44.

8. Lozupone, C.A., et al., *Diversity, stability and resilience of the human gut microbiota.* Nature, 2012. **489**(7415): p. 220-30.

9. Pellegrino, A., et al., *Role of Akkermansia in Human Diseases: From Causation to Therapeutic Properties.* Nutrients, 2023. **15**(8).

10. Wexler, A.G. and A.L. Goodman, *An insider's perspective: Bacteroides as a window into the microbiome.* Nat Microbiol, 2017. **2**: p. 17026.

11. Faith, J.J., et al., *The long-term stability of the human gut microbiota.* Science, 2013. **341**(6141): p. 1237439.

12. Antharam, V.C., et al., *Intestinal dysbiosis and depletion of butyrogenic bacteria in Clostridium difficile infection and nosocomial diarrhea.* J Clin Microbiol, 2013. **51**(9): p. 2884-92.

13. Sokol, H., et al., *Faecalibacterium prausnitzii is an anti-inflammatory commensal bacterium identified by gut microbiota analysis of Crohn disease patients.* Proc Natl Acad Sci U S A, 2008. **105**(43): p. 16731-6.

14. Machiels, K., et al., *A decrease of the butyrate-producing species Roseburia hominis and Faecalibacterium prausnitzii defines dysbiosis in patients with ulcerative colitis.* Gut, 2014. **63**(8): p. 1275-83.

15. El Aidy, S., et al., T*emporal and spatial interplay of microbiota and intestinal mucosa drive establishment of immune homeostasis in conventionalized mice.* Mucosal Immunol, 2012. **5**(5): p. 567-79.

16. Ouyang, Z.R., et al., *The role of short-chain fatty acids in Clostridioides difficile infection: A review.* Anaerobe, 2022. **75**: p. 102585.

17. Brown, E.M., et al., *Bacteroides-Derived Sphingolipids Are Critical for Maintaining Intestinal Homeostasis and Symbiosis.* Cell Host Microbe, 2019. **25**(5): p. 668-680 e7.

18. Otaru, N., et al., *GABA Production by Human Intestinal Bacteroides spp.: Prevalence, Regulation, and Role in Acid Stress Tolerance.* Front Microbiol, 2021. **12**: p. 656895.

19. Horvath, T.D., et al., *Bacteroides ovatus colonization influences the abundance of intestinal short chain fatty acids and neurotransmitters.* iScience, 2022. **25**(5): p. 104158.

20. Coulthurst, S., *The Type VI secretion system: a versatile bacterial weapon. Microbiology* (Reading), 2019. **165**(5): p. 503-515.

21. Russell, A.B., et al., *A type VI secretion-related pathway in Bacteroidetes mediates interbacterial antagonism.* Cell Host Microbe, 2014. **16**(2): p. 227-236.

22. Coyne, M.J., et al., *A family of anti-Bacteroidales peptide toxins wide-spread in the human gut microbiota.* Nat Commun, 2019. **10**(1): p. 3460.

23. Pellock, S.J., et al., *Three structurally and functionally distinct beta-glucuronidases from the human gut microbe Bacteroides uniformis.* J Biol Chem, 2018. ***293***(48): p. 18559-18573.

24. Wang, C., et al., *Protective effects of different Bacteroides vulgatus strains against lipopolysaccharide-induced acute intestinal injury, and their underlying functional genes.* J Adv Res, 2022. **36**: p. 27-37.

25. Hoces, D., et al., *Fitness advantage of Bacteroides thetaiotaomicron capsular polysaccharide in the mouse gut depends on the resident microbiota.* Elife, 2023. **12**.

26. Elahi, M., et al., *The Human Gut Microbe Bacteroides thetaiotaomicron Suppresses Toxin Release from Clostridium difficile by Inhibiting Autolysis.* Antibiotics (Basel), 2021. **10**(2).

27. Stentz, R., et al., *Cephalosporinases associated with outer membrane vesicles released by Bacteroides spp. protect gut pathogens and commensals against beta-lactam antibiotics.* J Antimicrob Chemother, 2015. **70**(3): p. 701-9.

28. Wang, K., et al., *Parabacteroides distasonis Alleviates Obesity and Metabolic Dysfunctions via Production of Succinate and Secondary Bile Acids.* Cell Rep, 2019. **26**(1): p. 222-235 e5.

29. Atarashi, K., et al., *Induction of colonic regulatory T cells by indigenous Clostridium species.* Science, 2011. **331**(6015): p. 337-41.

30. Greathouse, K.L., C.C. Harris, and S.J. Bultman, *Dysfunctional families: Clostridium scindens and secondary bile acids inhibit the growth of Clostridium difficile.* Cell Metab, 2015. **21**(1): p. 9-10.

31. Kang, J.D., et al., *Bile Acid 7alpha-Dehydroxylating Gut Bacteria Secrete Antibiotics that Inhibit Clostridium difficile: Role of Secondary Bile Acids.* Cell Chem Biol, 2019. **26**(1): p. 27-34 e4.

32. Matsushita, M., et al., *Firmicutes in Gut Microbiota Correlate with Blood Testosterone Levels in Elderly Men.* World J Mens Health, 2022. **40**(3): p. 517-525.

33. Wu, W., et al., *Protective Effect of Akkermansia muciniphila against Immune-Mediated Liver Injury in a Mouse Model.* Front Microbiol, 2017. **8**: p. 1804.

34. Lessa, F.C., et al., *Burden of Clostridium difficile infection in the United States.* N Engl J Med, 2015. **372**(9): p. 825-34.

35. Magill, S.S., et al., *Changes in Prevalence of Health Care-Associated Infections in U.S. Hospitals.* N Engl J Med, 2018. **379**(18): p. 1732-1744.

36. Guh, A.Y., et al., *Trends in U.S. Burden of Clostridioides difficile Infection and Outcomes.* N Engl J Med, 2020. **382**(14): p. 1320-1330.

37. Feuerstadt, P., N. Theriault, and G. Tillotson, *The burden of CDI in the United States: a multifactorial challenge.* BMC Infect Dis, 2023. **23**(1): p. 132.

38. Dubberke, E.R. and M.A. Olsen, *Burden of Clostridium difficile on the healthcare system.* Clin Infect Dis, 2012. **55 Suppl 2**: p. S88-92.

39. Vedantam, G., et al., *Clostridium difficile infection: toxins and non-toxin virulence factors, and their contributions to disease establishment and host response.* Gut Microbes, 2012. **3**(2): p. 121-34.

40. Nelson, W.W., et al., *Health care resource utilization and costs of recurrent Clostridioides difficile infection in the elderly: a real-world claims analysis.* J Manag Care Spec Pharm, 2021. **27**(7): p. 828-838.

41. Smits, W.K., et al., *Clostridium difficile infection.* Nat Rev Dis Primers, 2016. **2**: p. 16020.

42. Shields, K., et al., *Recurrent Clostridium difficile infection: From colonization to cure.* Anaerobe, 2015. **34**: p. 59-73.

43. Ma, G.K., et al., *Increasing Incidence of Multiply Recurrent Clostridium difficile Infection in the United States: A Cohort Study.* Ann Intern Med, 2017. **167**(3): p. 152-158.

44. Feuerstadt, P., et al., *Clinical burden of recurrent Clostridioides difficile infection in the medicare population: A real-world claims analysis.* Antimicrob Steward Healthc Epidemiol, 2022. **2**(1): p. e60.

45. Shahinas, D., et al., *Toward an understanding of changes in diversity associated with fecal microbiome transplantation based on 16S rRNA gene deep sequencing.* mBio, 2012. **3**(5).

46. Chang, J.Y., et al., *Decreased diversity of the fecal Microbiome in recurrent Clostridium difficile-associated diarrhea.* J Infect Dis, 2008. **197**(3): p. 435-8.

47. Seekatz, A.M., et al., *Dynamics of the fecal microbiome in patients with recurrent and nonrecurrent Clostridium difficile infection.* Genome Med, 2016. **8**(1): p. 47.

48. Schubert, A.M., et al., *Microbiome data distinguish patients with Clostridium difficile infection and non-C. difficile-associated diarrhea from healthy controls.* mBio, 2014. **5**(3): p. e01021-14.

49. Berkell, M., et al., *Microbiota-based markers predictive of development of Clostridioides difficile infection.* Nat Commun, 2021. **12**(1): p. 2241.

50. Manges, A.R., et al., *Comparative metagenomic study of alterations to the intestinal microbiota and risk of nosocomial Clostridum difficile-associated disease.* J Infect Dis, 2010. **202**(12): p. 1877-84.

51. Rea, M.C., et al., *Clostridium difficile carriage in elderly subjects and associated changes in the intestinal microbiota.* J Clin Microbiol, 2012. **50**(3): p. 867-75.

52. Finn, E., F.L. Andersson, and M. Madin-Warburton, *Burden of Clostridioides difficile infection (CDI) - a systematic review of the epidemiology of primary and recurrent CDI.* BMC Infect Dis, 2021. **21**(1): p. 456.

53. Theriot, C.M., A.A. Bowman, and V.B. Young, *Antibiotic-Induced Alterations of the Gut Microbiota Alter Secondary Bile Acid Production and Allow for Clostridium difficile Spore Germination and Outgrowth in the Large Intestine.* mSphere, 2016. **1**(1).

54. Theriot, C.M. and V.B. Young, *Interactions Between the Gastrointestinal Microbiome and Clostridium difficile.* Annu Rev Microbiol, 2015. **69**: p. 445-61.

55. Li, X., et al., *A strain of Bacteroides thetaiotaomicron attenuates colonization of Clostridioides difficile and affects intestinal microbiota and bile acids profile in a mouse model.* Biomed Pharmacother, 2021. **137**: p. 111290.

56. Theriot, C.M. and V.B. Young, *Microbial and metabolic interactions between the gastrointestinal tract and Clostridium difficile infection.* Gut Microbes, 2014. **5**(1): p. 86-95.

57. Ridlon, J.M., D.J. Kang, and P.B. Hylemon, *Bile salt biotransformations by human intestinal bacteria.* J Lipid Res, 2006. **47**(2): p. 241-59.

58. Ducarmon, Q.R., et al., *Gut Microbiota and Colonization Resistance against Bacterial Enteric Infection.* Microbiol Mol Biol Rev, 2019. **83**(3).

59. Aguirre, A.M. and J.A. Sorg, *Gut associated metabolites and their roles in Clostridioides difficile pathogenesis.* Gut Microbes, 2022. **14**(1): p. 2094672..

60. Thanissery, R., J.A. Winston, and C.M. Theriot, *Inhibition of spore germination, growth, and toxin activity of clinically relevant C. difficile strains by gut microbiota derived secondary bile acids.* Anaerobe, 2017. **45**: p. 86-100.

61. Sorg, J.A. and A.L. Sonenshein, *Bile salts and glycine as cogerminants for Clostridium difficile spores.* J Bacteriol, 2008. **190**(7): p. 2505-12.

62. Theriot, C.M., et al., *Antibiotic-induced shifts in the mouse gut microbiome and metabolome increase susceptibility to Clostridium difficile infection.* Nat Commun, 2014. **5**: p. 3114.

63. Sorg, J.A. and A.L. Sonenshein, *Inhibiting the initiation of Clostridium difficile spore germination using analogs of chenodeoxycholic acid, a bile acid.* J Bacteriol, 2010. **192**(19): p. 4983-90.

64. Sorg, J.A. and A.L. Sonenshein, *Chenodeoxycholate is an inhibitor of Clostridium difficile spore germination.* J Bacteriol, 2009. **191**(3): p. 1115-7.

65. Deng, H., et al., *Bacteroides fragilis Prevents Clostridium difficile Infection in a Mouse Model by Restoring Gut Barrier and Microbiome Regulation.* Front Microbiol, 2018. **9**: p. 2976.

66. Antharam, V.C., et al., *An Integrated Metabolomic and Microbiome Analysis Identified Specific Gut Microbiota Associated with Fecal Cholesterol and Coprostanol in Clostridium difficile Infection.* PLoS One, 2016. **11**(2): p. e0148824.

67. *REBYOTA [package insert]*. 2022, Ferring Pharmaceuticals Inc: Parsippany, NJ.

68. Chopra, T., *A profile of the live biotherapeutic product RBX2660 and its role in preventing recurrent Clostridioides difficile infection.* Expert Rev Anti Infect Ther, 2023. **21**(3): p. 243-253.

69. Khanna, S., et al., *Efficacy and Safety of RBX2660 in PUNCH CD3, a Phase III, Randomized, Double-Blind, Placebo-Controlled Trial with a Bayesian Primary Analysis for the Prevention of Recurrent Clostridioides difficile Infection.* Drugs, 2022.

70. Orenstein, R., et al., *Durable reduction of Clostridioides difficile infection recurrence and microbiome restoration after treatment with RBX2660: results from an open-label phase 2 clinical trial.* BMC Infect Dis, 2022. **22**(1): p. 245.

71. Dubberke, E.R., et al., *Final Results from a Phase 2b Randomized, Placebo-Controlled Clinical Trial of RBX2660: A Microbiota-Based Drug for the Prevention of Recurrent Clostridioides difficile Infection.* Infect Dis Ther, 2023. **12**(2): p. 703-709.

72. Blount, K., et al., *Significant and durable microbiome compositional changes and clonal engraftment in a phase 3 trial of fecal microbiota, live-jslm for recurrent clostridioides difficile infection,* in *In*: MAD-ID; May 10-13. 2023: Orlando, Florida.

73. Blount, K., et al., *Microbiome and bile acid restoration was consistent across three clinical trials of fecal microbiota, live-jslm for recurrent Clostridioides difficile infection: A combined analysis,* in *In*: MAD-ID; May 10-13. 2023: Orlando, Florida.

**Acknowledgment**, the authors thank Angela Donald ND, MSc for writing support.

**Conflict of Interest Statement:**

**Glenn Tillotson** is a consultant to Ferring Pharmaceuticals and Spero Therapeutics, **Gayatri Vedantam** is an employee of the School of Animal & Comparative Biomedical Sciences, University of Arizona, Tucson, AZ, USA, **Jae Hyun Shin** is an employee of the Division of Infectious Diseases & International Health, University of Virginia, Charlottesville, VA, USA, **Cirle A. Warren** is on the Medical Advisory Board for Seres Therapeutics and site-PI for Rebyota Prospective Registry.
